# The Long-Term Effects of the COVID-19 Pandemic on Children’s Writing: a Follow-up Replication Study

**DOI:** 10.1007/s10648-023-09729-1

**Published:** 2023-02-02

**Authors:** Gustaf B. Skar, Steve Graham, Alan Huebner

**Affiliations:** 1 Norwegian University of Science and Technology, Trondheim, Norway; 2 University of Gothenburg, Gothenburg, Sweden; 3 Arizona State University, Tempe, AZ USA; 4 Australian Catholic University – Brisbane, Sydney, Australia; 5 University of Notre Dame, Notre Dame, IN USA

## Abstract

The COVID-19 pandemic and the move by governments worldwide to cancel in-class instruction and move to emergency remote instruction in March and April of 2020 created an unprecedented disruption in children’s education. As the COVID-19 pandemic took form and continued to impact education in the following 2020/2021 academic year, multiple concerns were raised about possible negative effects on students’ learning. The current longitudinal replication study examined this proposition for second-grade students in Norway. In a previous investigation (Skar et al. Journal of Educational Psychology 114:1553–1566, 2022), we found that scores for quality of writing, handwriting fluency, and attitude toward writing of first-grade children tested immediately after emergency remote instruction ended in the Spring of 2020 (During COVID-19 cohort) were lower than the scores of first-grade students from the same schools tested a year earlier before the start of the pandemic (Before COVID-19 cohort). In the present study, we compared the scores for the During COVID-19 cohort (333 girls, 308 boys) on these same writing measures 1 year later at the end of second grade to a During COVID-19 cohort of second-graders (888 girls, 780 boys) from the same schools tested 2 years earlier before the start of the pandemic. The initial negative impact of the COVID-19 pandemic on first-grade students’ writing observed by Skar et al. (Journal of Educational Psychology 114:1553–1566, 2022) was no longer evident 1 year later at the end of second grade in the current study.

In December 2019, the SARS-CoV-2 virus quickly spread across the world, creating unprecedented economic and social challenges, impacting virtually all aspects of daily life, including educating children (Reimers, 2022). One strategy that countries used to slow the spread and impact of this virus involved moving in-person learning at schools to remotely delivered instruction (e.g., online, radio) or some combination of remote and in class instruction (Di Pietro et al., 2020; Hodges et al., 2020). Most countries implemented this strategy in March or April of 2020, impacting over 1.7 billion young people in schools and universities worldwide (UNESCO, 2020). As the pandemic evolved, some countries continued remote instruction into the next school year, others returned to in class instruction, and still others reinstituted remote instruction after in class instruction had been resumed (Azner et al., 2021; OECD, 2021). The interruption of in-class teaching in schools resulted in a loss of learning time for students (e.g., Huber et al., 2020; OECD, 2021) as well as concerns about the quality of teaching during remote instruction (e.g., Blikstad-Balas et al., 2022; Di Pietro et al., 2020).

The COVID-19 pandemic not only resulted in a shift to remote instruction for some period of time for most students worldwide, but it also influenced multiple aspects of schooling when classes did meet in schools in person. For example, social distancing, masks, and hand washing became common in many schools after students returned to classrooms following remote teaching (Esposito et al., 2021). This required rearranging teaching environments and adjusting instruction. COVID-19 further resulted in changes in working conditions for teachers, as many countries changed their school calendars and curriculum, while at the same time they recruited more temporary staff (OECD, 2021). As the pandemic progressed, teachers reported high levels of stress and emotionally exhaustion, which negatively impacted their sense of well-being (Chan et al., 2021). Students, parents, and teachers faced additional challenges as COVID-19 diminished family income and increased food insecurity, domestic violence, and mental health problems (UNESCO, 2020). The underlying uncertainty of the course of COVID-19, its observed effects on individuals and society, and the fear the pandemic generated created a context for some students “that undermined the necessary focus and dedication to schoolwork” (Reimers, 2022; p. 2).

Not surprisingly, there has been considerable concern about the impact of the COVID-19 pandemic on students’ learning (e.g., Daniel, 2020; OECD, 2021; UNESCO, 2020). Evidence to support such concerns have been quantified in two systematic reviews. In 11 studies, Hammerstein et al. (2021) found there was a median drop of − 0.10 *SD* for mathematics and − 0.09 *SD* for reading before and after remote instruction was implemented in March or April of 2020. Younger students and students from poor households were more negatively impacted by remote learning than older youngsters and students from more affluent families. In a second review published a year later, König and Frey (2022) examined the effects of remote instruction implemented during the Spring of 2020 or later. Collectively, the 18 studies reviewed resulted in a − 0.18 *SD* across all academic measures (mostly mathematics and reading). Although not statistically significant, the researchers indicated the effects of remote instruction tended to be more pronounced for younger than older students, and tended to be more impactful when remote instruction occurred earlier rather than later. Consequently, it is possible that the effects of the COVID-19 pandemic on student achievement become less pronounced over time, but this may depend on the type of students studied (see also De Witte & Smet, 2021; Harmey & Moss, 2021). Both of these issues were addressed in the current investigation.

## Study Purpose

The purpose of this study was to determine the longitudinal impact of the COVID-19 pandemic for children in first grade who received remote instruction in the Spring of 2020, but returned to in-class instruction the following year during second grade (referred to as the During COVID-19 cohort). More specifically, we determined if these Norwegian second-grade students’ performance on three writing measures (writing quality, handwriting fluency, and attitude toward writing) administered in May/June of 2021 differed from that of second-grade students from the same schools who had completed the same assessments in May/June of 2019 before the first case of the COVID-19 pandemic was reported (referred to as the Before COVID-19 cohort). We further examined whether possible learning losses for the During COVID-19 cohort were mitigated by gender and students’ primary language (native Norwegian speaker, bilingual speaker with Norwegian and at least one other language as a native language, and native speaker of a language other than Norwegian). The current investigation was a longitudinal replication of an earlier study conducted by Author Skar et al. (2022).

In the prior study by Author Skar et al. (2022), a Before COVID-19 group of 1636 Norwegian first-grade students completed assessments of writing quality, handwriting fluency, and attitude toward writing in May/June of 2019. The following year, a second cohort of 817 first-grade students from the same schools completed identical writing assessments in May/June 2020. This During COVID-19 cohort of first-graders completed these assessments just following the end of remote instruction due to COVID-19 in Norway. Students returned to in-class instruction on April 28, 2020. As a result, this prior investigation by Author Skar et al. (2022) compared the writing of cohorts of first-grade students attending the same schools who had and had not experienced remote instruction and COVID-19 pandemic conditions. The writing data for the Before COVID-19 cohort provided a benchmark for what students in the During COVID-19 cohort should have achieved.

Because no two groups of students in the same schools are exactly alike from one year to the next, Author Skar et al. (2022) controlled for variance due to both school and student variables when examining writing differences between the Before and During COVID-19 first-grade cohorts. This included school size, school performance on national tests, proportion of certified teachers, number of students per special education teacher, average school hours teaching students, nesting of classes and schools, student gender, and students’ primary language. School variables like these are related to students’ writing performance (Walberg & Ethington, 1991), as are student variables such as gender (Ekholm et al., 2018; Graham et al., 1998; Reilly et al., 2019) and language (Camping et al., 2020; Corderio et al., 2018).

Skar et al. (2022)) found that the During COVID-19 cohort of first-grade Norwegian students had statistically significant lower scores on the three writing measures than the Before COVID-19 cohort of first-graders after variance due to school and student characteristics were controlled. The negative effects of remote instruction were most pronounced for writing quality and handwriting fluency, representing small to medium effects. The effect for attitude toward handwriting was also negative, but small and less pronounced than the effects for the other two writing outcomes. In addition, students learning to speak Norwegian evidenced more negative COVID-19 outcomes than native Norwegian speakers on the handwriting fluency and writing quality measures, whereas the quality of bilingual students’ writing was more negatively impacted by COVID-19 than the writing quality of their native Norwegian speaking peers. Finally, boys evidenced stronger negative effects than girls on all three measures of writing.

The current study was virtually identical to the Skar et al. (2022) investigation. It was conducted in the same schools, administered the same writing assessments, controlled variance for identical school and student variables, and applied the same research design to assess the possible effects of the COVID-19 pandemic. The During COVID-19 students in the present study also participated in the earlier investigation as first-grade students, but they were now second-graders. The second-grade Before COVID-19 students in this study were new, however. All Before COVID-19 students in the prior investigation were in first grade. Skar et al. 2022 examined the effects of remote instruction and COVID-19 just after Norwegian first-grade students returned to classes in person, whereas the current study examined these effects 1 year later as these same students were close to ending second-grade.

## Research Question and Prediction

The present study answered the following research question: Did the COVID-19 pandemic instruction negatively impact the quality of second-grade students’ writing, handwriting fluency, and attitude toward writing 1 year after the pandemic began?

Most of the research examining the effects of COVID-19 on students’ academic outcomes has focused on mathematics and reading (see De Witte & Smet, 2021; Hammerstein et al., 2021; König & Frey, 2022; OECD, 2021). Besides the Skar et al. (2022) investigation with first-graders in Norway, we only located one additional study examining the effects of the COVID-19 pandemic on writing. This was a study by Haelermans et al. (2021) with primary grade children in the Netherlands, showing a COVID-19-related drop in spelling performance on a standardized test of − 0,06 *SD*.

It is especially important to examine the effects of the COVID-19 pandemic on young students’ writing. Writing is a powerful tool for communicating, persuading, informing, and entertaining others, whereas writing about text read and material presented in class enhances learning (Graham et al., 2018; Graham et al., 2020). If the COVID-19 pandemic results in sustained learning losses for beginning writers, the consequences may reverberate across the school years. There is a general consensus in the education community that problems that occur in the earliest grades, if not corrected quickly, become more problematic with time (Slavin et al., 1989).

The theoretical model that guided the prior Skar et al. (2022) investigation and the current longitudinal replication was the Writer(s)-within-Community model (WWC; Graham, 2018a, 2018b). The WWC proposed that writing development is simultaneously and interactively shaped and bound by where it is learned and the cognitive capabilities and resources of those learning to write. This influenced our decision in this and the previous study by Skar et al. (2022) to focus on a single country (Norway). The teaching of writing in any single country is influenced by its own unique cultural, social, institutional, political, and historical factors (Graham, in press) as was each country’s response to the COVID-19 pandemic (Reimers, 2022).

The WWC model also affected our decisions on which aspects of writing to assess. The model proposed that students’ motivations for writing, including attitude toward writing, fuel effort and provide the impetus for students to apply available cognitive resources to write. These cognitive resources include executive functioning processes to regulate writing production processes involving conceptualization, ideation, translation, transcription, and reconceptualization. For beginning writers, their cognitive resources are limited and transcription skills such as handwriting are so slow and effortful they interfere with other writing production processes like conceptualization and ideation (Graham et al., 1997). Consequently, we assessed students’ attitude toward writing and handwriting fluency along with the overall quality of their writing (i.e., the end product of applying cognitive resources to write).

We predicted that the learning loss in writing quality, handwriting fluency, and attitude toward writing observed in the Skar et al. (2022) first-grade study would still be evident in second grade, but to a lesser degree. While the Norwegian government did not universally require that all schools again move to remote instruction as happened in the Spring of 2020, some schools did cancel in class instruction for a short period of time. Even when students were at school in person, the COVID-19 pandemic continued to impact teachers, students, and family in multiple ways that could negatively impact learning. As noted earlier, this included instructional adjustments in response to social distancing and teacher absences, higher levels of teacher stress and emotional exhaustion, diminished family income and greater food insecurity, and increased domestic violence and mental health problems, as well as the uncertainty and fear caused by the pandemic (Esposito et al., 2021; OECD, 2021; Reimers, 2022; UNESCO, 2020). Further, the Norwegian government did not increase educational funding, implement special educational policies, increase national efforts to help teachers, use standardized assessments to track possible learning losses, or provide incentives at the national level for teachers to provide remedial classes during 2020/2021 as mechanisms for counteracting the effects of the pandemic, which other countries did implement (Blikstad-Balas et al., 2022; De Witte & Smet, 2021; Reimers, 2022).

Even so, there were multiple factors operating in Norway that could potentially weaken the impact of the COVID-19 pandemic on young students’ writing over time. The length of remote instruction in Norway was relatively short. In the primary grades, Norwegian schools were only closed for in-person instruction for 29 school days during 2020 and 2021 versus 78 school days on average for all OCED countries (OCED, 2021). Thus, the possible negative effects of the rapid shift to distance learning in most countries in the Spring of 2020 to combat COVID-19 (see Di Pietro et al., 2020) may be more limited in Norway than other countries that relied on remote learning for longer periods of time or employed this tactic more often. There are several protective factors that may further reduce the long-term negative effects of the pandemic on young Norwegian students’ writing. Norway is an affluent country with a strong educational system where teachers have considerable autonomy to make decisions about instruction (Blikstad-Balas et al., 2022). This may have made it easier for teachers to positively adjust their instruction to meet the new realities of the pandemic. Finally, the meta-analysis by König and Frey (2022) suggested that learning loss at the start of COVID-19 was larger than learning loss measured 1 year or later into the pandemic. In the case of the current study, the potential of smaller learning losses in writing for students at second grade may be off-set by König and Frey’s observation that learning loss tended to be higher for younger than older students.

## Methods

### Setting

In response to concerns about COVID-19, Norway canceled in class instruction and implemented a model of emergency remote instruction beginning March 12, 2020, and ending April 27, 2020. While the Norwegian government did not move *all* schools to remote instruction again, individual schools could move to remote instruction for a limited time if certain conditions were met.

At the time of this investigation, there were two information sources regarding measures taken by schools to hinder the spread of the SARS-CoV-2 virus. Both sources reported data at the school level only, and we did not have access to individual level data for this investigation. One source was the “Grunnskolens informasjonssystem” (*The Information System of Grade 1–10 School*). It reported the number of absent teachers and students in week 39 of 2020 (i.e., four weeks into the academic year of 2020–2021). The average sick leave rate across schools nationwide was 18.4%. It also reported whether schools had applied special disease-hindering measures according to a model specified by the Norwegian Directorate for Health (NDH). The NDH model^1^ specified three levels of measures signaled by the following colors. Green indicated more or less business as usual, but sick students, teachers, and other personnel were not allowed on the school premises and physical contact among individuals should be avoided. Yellow specified that employees should keep at least 1 m (3′ 3″) distance at all times, and that students of different groupings were not allowed to have contact. Red meant that classes would be reduced, and schools should take measures to avoid any large gathering of students and staff. No schools in our sample applied the red level. This was done in 2% of schools in Norway nationwide. However, the average sick leave rate among teachers in our sample for the During COVID-19 cohort was 22.7% (higher than the national average of 18.4%).

The second information source was the “Konsekvenser av smitteverntiltak i grunnskolen – våren 2021” (*Consequences of measures taken to hinder disease spread—spring 2021*) (Norwegian Directorate for Education & Training, 2021), which summarized the situation in the period between January 4 and March 12 in 2021. The main findings were that 25% of schools were closed at some point during that period, that 25% of teachers and 16% of students were home sick in week 10 of 2021. There was no way to obtain these statistics for individual schools, but these findings, in sum, suggest that schools continued to struggle with handling the spread of the SARS-CoV-2 virus.

### Participants

Participants were 2309 students in second grade in Norway. Of these students, 1668 attended second-grade 1 year prior to the COVID-19 pandemic outbreak (academic year 2019/2020) and are referred to as the Before COVID-19 cohort. The other 641 students attended second-grade 1 year into the pandemic (academic year 2020/2021) and are referred to as the During COVID-19 cohort. All students in the During COVID-19 cohort were first-grade participants in the Skar et al. (2022) investigation.

The students in the current study came from 185 classrooms in 59 schools in four municipalities. Two municipalities represented major urban areas, whereas the other two municipalities represented more rural areas. For the Before COVID-19 cohort, the average school size was 477 (*SD* = 172), and for the During COVID-19 cohort the average school size was 441 (*SD* = 171). The average number of instructional hours divided by number of students was 54.4 h (*SD* = 11.5) for the Before COVID-19 cohort, and it was 55.4 h (*SD* = 8.89) for the During COVID-19 group. The proportion of certified teachers was 96.3 (*SD* = 5.33) and 95.7 (*SD* = 4.84), respectively, for the Before COVID-19 and During COVID-19 cohorts. The number of students per special education assistants were 86.1 (*SD* = 63.1) for the Before COVID-19 cohort, and it was 91.1 (*SD* = 31.1) for the During COVID-19 cohort. Lastly, in terms of school characteristics, the average score on national tests for schools was 54.1 (*SD* = 2.84), and 50.4 (*SD* = 2.17) for the Before and During COVID-19 cohorts, respectively.

There were 888 girls (53.2%) in the Before COVID-19 cohort, and 333 girls (51.9%) in the During COVID-19 cohort. The differences in proportions were not statistically significant (*χ*^2^(1) = 0.258, *p* = 0.611). Furthermore, the Before COVID-19 cohort included 1388 (83.2%) students with Norwegian as their first language (the L1 group), 98 students (5.8%) with Norwegian as their second language (the L2 group), and 182 (10.9%) students who had Norwegian and one or several additional languages as their first language (the bilingual group). For the During COVID-19 cohort, there were 508 (79.3%) students in the L1 group, 41 (6.4%) students in the L2 group, and 92 (14.4%) students in the bilingual group. A chi-square test of independence indicated that there was no statistical difference between the Before and During COVID-19 cohorts in terms of proportions of students in the L1, L2, and bilingual groups (*χ*^2^(2) = 5.715, *p* = 0.057). It was not possible in Norway for us to systematically collect information about what language other than Norwegian participating children spoke.

Data that was available to us about the participating children and schools suggests that they were representative of second-grade children nationwide. First, school scores for the national tests in English, mathematics, and reading administered to fifth-graders were 51.4 for the Before COVID-19 cohort and 50.4 for the During COVID-19 cohort. This compared favorably to the national average of 50.0.

Second, the gender proportion in our sample was similar to the proportions nationally. In the academic year of 2018–2019, 48.6% of second-grade students in Norway were girls, which is just below the 95% confidence interval (50.8–55.7%) for the proportion of girls in the Before COVID-19 cohort. In the academic year 2020–2021, 49.1% of the second-grade population was girls, which is within the 95% confidence interval (48–55.9%) for the proportion of girls in the During COVID-19 cohort.

Third, while Norway lacks publicly available data on the language backgrounds of students, there are available indices on the proportion of students entitled to extra-curricular language instruction. In 2018–2019 and in 2020–2021, the percentages of second-graders who received such extra-curricular instruction were 8.8% and 8.7% respectively. These proportions are similar to the proportions of students in the L2 groups of our sample, although they are both marginally outside or adjacent to the 95% confidence interval for the proportion L2 speakers in the Before COVID-19 cohort (4.8–7.1%) and in the During COVID-19 cohort (4.7–8.7%).

Fourth, the municipalities from which our sample of students were drawn are generally representative of other municipalities in Norway. The four municipalities represented in this investigation were two large, one average, and one small municipality. The largest municipality, containing both urban and rural areas, had a population of 697,010 (12.9% of Norway’s 5,391,369 residents), and the smallest municipality had a population of 7077 (0.01% of Norway’s residents).

Lastly, our two cohorts were similar to national averages on three other measures. The proportion of certified teachers across the Before and During COVID-19 cohorts (96.2%) both groups was comparable to the national average (95.2%). The average number of school hours per student was 54.8 in our two cohorts collectively compared to 61 h nationally. The average number of students per special education teacher for our cohorts was 87.5 (*SD* = 34.8) and 83.8 (*SD* = 98.4) nationally.

### Sampling Procedures

All students in this study were initially recruited to participate in a large-scale RCT writing intervention study (Skar, Aasen et al., 2020), lasting between 2019 and 2021. Recruitment for participation in the RCT study took place at the school level, and was aided by executive officers in the different municipalities. Schools were randomly assigned to either the writing treatment or control conditions.

The second-grade students in the Before COVID-19 cohort participated in a one-off testing administration in May/June 2019 before schools were randomly selected to participate in either an intervention or a control group in the RCT study. We approached 2276 students before testing, and 2076 (91.2%) consented to participation through signatures by parents/guardians. This was reduced to 1668 students once children were eliminated who had not completed the writing assessments (19.7% or 408 children). At that point, none of the Before COVID-19 students had received any form of writing intervention from our research group. Further, the students who were eventually in the writing treatment and control schools did not evidence statistically different scores for handwriting fluency, text quality, and attitude toward writing measures.^2^

In order to eliminate possible confounding effects from receiving a writing intervention in the RCT study, all second-grade students in the During COVID-19 cohort in this investigation were from schools assigned to the control condition in the RCT. All second-grade students in this cohort were also first-grade participants in the During COVID-19 cohort in the earlier study by Skar et al. (2022). This sampling strategy ensured comparability of findings from this and the prior study. Initially, we approached 1343 second-grade students for possible participation in the During COVID-19 cohort, and 1139 (84.8%) consented to participate (also by signatures by parents/guardians). This was reduced to 641 students once children were eliminated who had not participated in the earlier Skar et al. (2022) investigation (3.1% or 25 children) or were missing writing assessments (18.5% or 151 children). Appendix A provides a graphical organizer outlining the participant flow.

It must be noted that the sample size for the Before COVID-19 group was 2.6 larger than the During COVID-19 group. This occurred for two reasons. One, we did not include students in the During COVID-19 group from schools that had received a writing treatment as part of the RCT study. As indicated earlier, this would have biased any comparison between the Before and During COVID-19 groups. Two, we did not include 25 students in the During COVID-19 group who were not participants as first-graders in the earlier Skar et al. (2022) investigation examining the effects of COVID-19. Also, as indicated earlier, this ensured comparability between the findings from the current and earlier investigation. While multiple imputation could have increased the sample size for the During COVID-19 group, only 151 students were removed from this group due to missing data. As a result, we decided not to use multiple imputation to calculate missing data in the current study because the use of such procedures with the three-level statistical model applied (see the “Analytical Strategy” section) creates technical issues that are not easily overcome, and multiple imputation would not have substantially reduced differences in sample size between the Before and During COVID-19 groups.

### Writing Measures and Covariates

Writing performance was measured with three tasks: a copy task, a discursive writing task, and a questionnaire. The results of the tasks were used to provide measures of handwriting fluency (copy task), writing quality (discursive writing task), and attitude toward writing (questionnaire). Both groups (Before and During COVID-19) were administered the same tasks. The analyses of differences in the writing scores of the Before and During COVID-19 cohorts also included eight covariates. All measures and covariates are described below.

### Handwriting Fluency

The copy task was taken for the Group Diagnostic Reading and Aptitude and Achievement Tests (Monroe & Sherman, 1996) and, in accordance with previous investigations (Graham et al., 1997), prompted students to as quickly and as accurately as possible copy a paragraph of text in 1.5 min. The number of letters that students copied correctly was divided by 1.5 to derive a measure of letters per minute, with the ensuing number serving as an estimate of handwriting fluency.

The copying task was administered by the students’ teacher who used a video to introduce the task. The video informed students the teacher was going to read a paragraph aloud, and they were to copy as much as possible of the paragraph in a 90-s interval. The video also instructed students to start and stop copying at the signal of the teacher.

Trained coders entered the number of correct letters into a spreadsheet. Letters that were correct but did not match the text were not counted, nor were incorrectly written letters or skipped letters. Ten percent of the material was double coded for the purpose of estimation of reliability, which was good (κ = 0.812, ICC = 0.99).

### Writing Quality

To elicit students’ discursive writing, students were prompted to write a response letter to the researchers at a Norwegian university. The prompt was developed in the abovementioned RCT, and it has been administered to 8000 + students in grades 1–3 in Norway. The writing tasks asked students to write a letter telling researchers at the Norwegian university what they enjoyed doing during recess time.

The discursive writing task was administered by students’ teachers. A video along with printed instruction on how to administer the writing tasks was sent to teachers. Teachers were asked to first engage students in a discussion about activities the students engaged in at recess time, with the purpose of orally generating content that could be used when writing. Teachers were further asked to project an image of children playing at a playground/school yard, to further spark ideas among students about what to write in the letter. Teachers discussed the purpose of the written response (to share information with researchers) as well as the typical format of a letter. Students were given a whole period (i.e., 45 min) to write the letter, as this follows normal procedures for conducting similar activities in Norwegian schools. Teachers were asked not to help students as they completed the writing task.

When students had finished writing their letters, their written responses were sent to the university of the first author where they were masked (i.e., stripped of information about name, school, age, gender, or anything else that could be considered to inform a rater about the student having written the text). Student texts were then scored by a group of trained raters who assessed each text on eight separate writing assessment scales: audience awareness, organization, content relevance, vocabulary, sentence construction, spelling, legibility and punctuation. For each writing assessment scale, a rater assigned a value between 1 and 5, with 5 indicating most quality. The score for this measure was the average score for all eight scales.

The scales were developed to capture important aspects of text quality. As an example, consider *audience awareness* which targeted the extent to which a text not only communicated with the reader, but also if the young author had managed to decontextualize his/her writing enough for a reader to make meaning of the content even if the reader was unable to clarify content in interviews with the author (Skar, Aasen et al., 2022). Prior to this investigation, the rating scales had been validated (Skar, Jølle, et al., 2020) and used to assess thousands of texts (Ska, Lei, et al. 2022). Please refer to Appendix A in Skar, Kvistad, et al. (2022) for descriptors for all eight scales.

Each student text was rated individually by two trained raters. Rater training consisted of an introduction to understanding the rating scales, and a trial round of assessing texts individually with ensuing group discussions. Accompanying each rating scale were training materials. These materials included annotated student texts, with annotations illustrating scoring levels for all scales. For practical reasons, students’ texts were rated on two occasions: immediately after data collection in 2019 (Before COVID-19 cohort), and immediately after data collection in 2021 (During COVID-19 cohort). Twenty-five researchers and graduate students formed the rater pool on the first occasion, and 24 researchers and graduate students formed the rater pool on the second occasion. Nine raters from the first occasion participated in the second occasion. To develop a score equation, 50 texts from the first occasion were rated also at the second occasion.^3^

Ratings were fitted to the following “many-facet Rasch measurement” (MFRM) model (Linacre, 2018a, 2018b):

$$loglog \left[{P}_{nij(k)}/{P}_{nij(k-1)}\right] ={B}_{n}-{E}_{i}-{C}_{j}-{F}_{x}$$

where ${P}_{nij(k)}$ represented the probability of student *n*, rated on rating scale $i$, by rater $j$, receiving a score of $k$, and ${P}_{nij(k-1)}$ represents the probability of the same student under the same conditions receiving a score of $(k-1)$. ${B}_{n}$ was the ability for person $n$, ${E}_{i}$ was the difficulty of rating scale $i$, and ${C}_{j}$ is the severity of rater $j$. ${F}_{x}$ represented the intersection where category $k$ and $(k-1)$ were equally probable. As mentioned, estimates from the second occasion were comparable to estimates from the first occasion, since parameter estimates for 50 common texts were used as anchors. The MFRM analysis yielded a “fair average score,” for each student. This fair average score was the average across all ratings scales, adjusted for rater harshness.

The data-to-model-fit was assessed to be adequate. The “reliability of separation” (a Rasch analog to Cronbach’s alpha) was 0.94 for student texts from the Before COVID-19 cohort and 0.95 for the During COVID-19 cohort. The adequate fit was also indicated by the proportion of standardized residuals being equal to or exceeding 2 and 3: For the Before COVID-19 cohort, the standardized residuals ≥ 2 and ≥ 3 was 3.90% and 0.59%, respectively. For the During COVID-19 cohort, 4.97% standardized residuals were equal to or exceeding 2, while 0.49% were equal to or exceeding 3. As a rule of thumb, standardized residuals ≥ 2 should not be more than 5%, and standardized residuals =  > 3 should not be more than 1% (Eckes, 2011).

The MFRM analysis yielded a single, scaled score (from 1 to 5), which—in the lingo of the FACETS software used (Linacre, 2018a, 2018b)—was the “*fair* average” or a score generated to compensate for differences among raters’ harshness and the difficulty of rating scales.

### Attitude Toward Writing

To determine attitude toward writing, students completed a survey with the following four items: “I liked the writing task,” “I am satisfied with my text,” “I am satisfied with my effort,” and “I like to write.” Students rated the statements using a star system: three stars indicated most agreement, and one star indicated least agreement. The attitude score was derived by averaging the scores on each individual item. An exploratory factor analysis indicated that the four questions formed a single factor, accounting for 53.8% of the variance (coefficient alpha = 0.71).

### Covariates

We included the following school-level covariates in the analyses: national test result, school size, proportion of certified teachers, students per special education teacher, and school hours per student. Student-level covariates were gender and language background. At the student level, we also included group membership variable (Before COVID-19 cohort and During COVID-19 cohort). We collected data on gender and language background by asking students’ teachers to indicate gender and if the student had learned Norwegian first (denoted “L1”), another language than Norwegian first (denoted “L2”) or Norwegian and another language (denoted “bilingual”).

Table 1 presents the descriptive statistics for student level measures. For 35 students, there were no school-level variables made public, but rather than excluding these students we used mean imputation as the proportion of missing data was low.

**Table 1:** Descriptive statistics for student level measures

	Before COVID-19		During COVID-19	
Characteristic	*N*	*M (SD)*	*N*	*M (SD)*
HW fluency				
Bilingual boys	87	27.4 (11.3)	53	23.4 (10.8)
Bilingual girls	95	31.3 (14.7)	39	26.1 (9.3)
L1 boys	645	26.2 (10.5)	234	22.5 (9.5)
L1 girls	743	31.0 (11.7)	274	28.2 (11.9)
L2 boys	48	24.8 (9.2)	21	22.3 (8.9)
L2 girls	50	29.1 (14.2)	20	20.4 (9.2)
Text quality				
Bilingual boys	87	3.1 (0.5)	53	2.9 (0.6)
Bilingual girls	95	3.3 (0.5)	39	3.3 (0.5)
L1 boys	645	3.0 (0.5)	234	3.1 (0.5)
L1 girls	743	3.4 (0.5)	274	3.4 (0.5)
L2 boys	48	3.1 (0.4)	21	3.0 (0.5)
L2 girls	50	3.2 (0.4)	20	3.1 (0.4)
Attitude writing				
Bilingual boys	87	2.5 (0.5)	53	2.3 (0.6)
Bilingual girls	95	2.6 (0.4)	39	2.5 (0.4)
L1 boys	645	2.3 (0.5)	234	2.3 (0.5)
L1 girls	743	2.6 (0.4)	274	2.5 (0.4)
L2 boys	48	2.5 (0.5)	21	2.4 (0.6)
L2 girls	50	2.5 (0.5)	20	2.7 (0.3)

## Procedures

As described in Skar et al. (2022), the circumstances for data collection were adapted to the Norwegian context. There are few tests in Norwegian schools, and formal grades are not introduced until the eighth year of schooling. Moreover, the age at which students start school has for a number of years been subject to public debate. In July 2020, the Oslo Metropolitan University was commissioned to investigate how the current starting age (6 years) affects students,^4^ and in a newspaper article the PI of this project stated that “many are devastated and report about young children experiencing enormous pressure.”^5^ The students in this study were young, and because we did not want children, teachers, or guardians to get the impression that students participated in a high stakes testing situation, we asked teachers to administer all tasks.

To standardize data collection as much as possible, we provided teachers with extended test administration manuals detailing how much time to spend on each task, how to prepare students, how to monitor the testing situation, and how to assemble student responses. After consulting with teachers, the first author and colleagues decided the discursive writing task and the subsequent attitude toward writing survey would be administered during a regular school hour (60 min) and that students would be offered 45 min to complete the writing task and the survey. The copy task was also administered by teachers during school time, but took about 10–15 min with preparation, task fulfillment, and assembly.

We counterbalanced the task administration: half of the teachers administered the letter writing task and attitude toward writing measure first and the copying task second, whereas the other half did the opposite. The data was collected on two occasions. The Before COVID-19 cohort were tested May/June of 2019. The During COVID-19 cohort were tested May/June of 2021. On both occasions, teachers had a 15-day window to complete data collection.

### Analytical Strategy

Given the clustered, or nested, nature of the data (i.e., students within classrooms and classrooms within schools), multilevel linear regression (e.g., Snijders and Bosker, 2012) was used for the analyses. Specifically, each of the three writing measures (text quality, handwriting fluency, and attitude toward writing) was analyzed separately (i.e., each was used as the dependent variable in its own three-level regression analysis). For each measure, a random-intercept null model with no predictors was fit to calculate the intraclass correlation coefficients (ICCs), which indicate the correlation structure of the data. Three-level models result in two ICCs: an ICC for the third level (school) and an ICC for level 2 nested within level 3 (classrooms in schools). Then, for each measure, a random-intercept model was fit with the covariates described above. The five school-level covariates described above are all numerical and were standardized in the model, so the interpretations of their slopes are on a standard scale.

Students were included in the analyses only if they had observations for all three writing measures; thus, students with missing data on at least one writing measure were excluded from the analysis. This was done to ensure comparability of result across outcome measures. All analyses were performed using the lme4 package (Bates et al., 2015) in the R statistical software environment (R Core Team, 2020).

## Results

The first two rows of Table 2 display the two ICCs for each dependent variable. Text quality had the highest ICCs at each level. Thus, text quality was the most highly correlated within classes and schools. Specifically, the estimated correlation for text quality between two randomly selected students in the same school was 0.136, while the estimated correlation for text quality between two randomly selected students in the same classroom was 0.255. The correlations for handwriting fluency were smaller than those for text quality, but they were non-negligible. The correlations for attitude were far smaller than the correlations for the other two measures. While the ICC described the correlation structure, the metric ${R}^{2}$ indicated the amount of variance in the response explained by the covariates. In other words, ${R}^{2}$ was a measure of the predictive or explanatory power of the model. The third and fourth rows of Table 2 showed, respectively, the ${R}^{2}$ value of the model with all the covariates and the ${R}^{2}$ value of the model without the cohort covariate. Thus, the difference of those two ${R}^{2}$ values indicated that the contribution of the cohort covariate (Before COVID-19 cohort vs During COVID-19 cohort), which is shown in the last row of the table. The effect size ${f}^{2}= \frac{{R}^{2}}{1-{R}^{2}}$ was given in parentheses; an intuitive interpretation for ${f}^{2}$ is that values of 0.02, 0.15, and 0.35 represent small, medium, and large effects (Lorah, 2018). Thus, the cohort covariate is well below the small effect threshold for all three outcome measures.

**Table 2:** ICCs and effect size for dependent variables

	Measure		
Quantity	Text quality	Handwriting fluency	Attitude
ICC (class)	0.255	0.183	0.066
ICC (school)	0.136	0.090	0.010
*R*^2^, model with cohort membership (*f*^2^)	0.129 (0.148)	0.071 (0.076)	0.052 (0.055)
*R*^2^, model without cohort membership (*f*^2^)	0.126 (0.144)	0.065 (0.070)	0.051 (0.054)
*R*^2^, difference (*f*^2^)	0.003 (0.003)	0.006 (0.006)	0.001 (0.001)

Table 3 presents the regression parameter estimates and corresponding *p*-values for the covariates in the regression models for each outcome measure. Regarding the school-level covariates, national test scores were a statistically significant predictor of text quality, but it did not statistically predict handwriting fluency and attitude toward writing. On the other hand, school size was a statistically significant predictor for handwriting fluency, but not for the other two writing outcomes. Regarding the student-level covariates, girls scored significantly higher on average than boys on all three outcome measures. In addition, the During COVID-19 cohort scored on average 1.707 points lower than the Before COVID-19 group on handwriting fluency, which was not quite statistically significant at the 5% level. Differences between the During and Before COVID-19 cohorts for text quality and attitude toward writing were also not statistically significant.

**Table 3:** Regression parameter estimates and p-values in regression models for each outcome

	Outcome measure					
	Text quality		Handwriting fluency		Attitude writing	
Parameter	Estimate	*p*-value	Estimate	*p*-value	Estimate	*p*-value
Intercept	3.015	< 0.001	26.239	< 0.001	2.362	< 0.001
Nation test	0.084	0.013	0.969	0.126	0.015	0.421
School size	0.028	0.373	1.426	0.018	0.003	0.876
Prop. cert	0.002	0.959	0.207	0.739	0.003	0.866
Special	− 0.081	0.008	− 0.822	0.148	− 0.006	0.726
School hours	− 0.049	0.200	0.051	0.944	0.009	0.688
Gender						
Ref level boy						
Girl	0.312	< 0.001	4.731	< 0.001	0.233	< 0.001
Language						
Ref level L1						
Bilingual	− 0.080	0.009	− 0.700	0.337	0.009	0.778
L2	− 0.083	0.050	− 2.039	0.042	0.044	0.313
During COVID-19 cohort	0.074	0.089	− 1.707	0.055	-0.044	0.155

## Discussion

The SARS-CoV-2 virus led to the largest all-time worldwide disruption in education (e.g., Azoulay, 2020; Winthrop, 2020). Across the globes, most countries moved in-person instruction at school to remote instruction in March or April of 2020. This sudden cancelation of in-person instruction presented an extraordinary challenge for teachers and schools as learning was quickly moved online in most countries (Fauzi & Khusuma, 2020). While digital learning platforms can enable and support learning in multiple ways, the COVID-19 pandemic and the ensuing move to in-person school closures occurred at a time when many teachers were not ready to apply these tools effectively. For instance, just prior to the onset of the pandemic, many teachers in a study of OECD countries reported they needed additional training to use digital tools effectively and almost one-half of them noted students were not allowed to use such tools in the classroom (Schleicher, 2020). Moreover, the move to remote instruction appeared to result in a loss of learning time in many countries (Huber et al., 2020), including in Norway where the current study took place (Blikstad-Balas et al., 2022), and parents served as proxy educators in many households during remote instruction tasked with assisting their children’s learning (Di Pietro et al., 2020). As a result, multiple organizations predicted remote instruction would negatively impact students’ learning (Daniel, 2020; Schleicher, 2020), and it seemed likely that the longer and more often remote instruction occurred during the COVID-19 pandemic, the greater impact it would have on students’ learning.

The possible negative effects of the COVID-19 pandemic on students’ learning were not limited just to the use of remote learning to keep schools open, but to the possible impact of the virus on teaching and learning when schools were open for in class instruction. Instruction and classroom environments had to be adjusted to accommodate social distancing and masking mandates (Esposito et al., 2021). Teachers’ working condition were further modified in many countries as school calendars and curriculum were modified in response to the effects of the pandemic on schools (OECD, 2021). Teachers’ job became more challenging, and they reported high levels of stress and exhaustion (Chan et al., 2021). The context for student learning was further diminished by the pandemic, as it created a less positive atmosphere for learning at school and home for many children (Reimers, 2022). Additionally, students, teachers, and parents faced unwanted challenges related to a decrease in family income and increase in food insecurity, domestic violence, and mental health problems brought about by the effects of COVID-19 (UNESCO, 2020).

In an initial study conducted just after remote instruction ended in Norway in April of 2020, we found that remote instruction had a negative impact on first-grade students writing (Skar et al., 2022). First-grade students who completed assessments on the quality of their text, handwriting fluency, and attitude toward writing once remote instruction was terminated in Norway scored lower on all three of these measures than first-grade students in the same school who completed these assessments 1 year earlier before the start of the pandemic. A similar finding for spelling performance was found in the Netherlands for primary grade students by Haelermans et al. (2021). Likewise, two systematic reviews examining students learning more broadly found that remote instruction had a negative impact on students’ learning (Hammerstein et al., 2021; König & Frey, 2022). Consequently, the accumulated evidence supports the thesis that remote instruction implemented as a result of the COVID-19 pandemic resulted in students making less educational progress than normal.

What is not clear at this point in time is whether the initial learning loss that occurred as a result of emergency remote instruction has diminished, remained steady, or increased as the pandemic has continued. König and Frey (2022) in their meta-analysis of 18 investigations reported that studies conducted later during the pandemic (Fall and Spring, 2021) tended to exhibit less learning loss than those conducted earlier (end of Spring 2020). They further indicated that younger students tended to experience greater learning loss than older students as a result of the pandemic. Neither of these trends were statistically significant, however.

In the current study, we examined the effects of COVID-19 on the writing of Norwegian second-grade students at the end of the 2020/2021 school year. This was a little more than 1 year into the pandemic. Our primary focal group, the During COVID-19 cohort, had experienced remote instruction during March/April of 2020 and in-person instruction in school during all or most of second-grade during the continuing pandemic (a quarter of Norwegian schools had a short return to remote instruction). As in Skar et al. (2022), their performance on measures of writing quality, handwriting fluency, and attitude toward writing were compared to a cohort of same grade peers in the same schools at the end of the 2019 school year before SARS-CoV-2 virus existed (Before COVID-19 cohort). To help ensure that these two cohorts were as similar as possible, we controlled for variance due the nested nature of the data (students within classrooms and classrooms within schools), school variables (school size, proportion of certified teachers, students per special education teacher, school hours per student, and national test results for fifth-grade students in schools), and student variables (gender and students’ language [L1, bilingual, L2]). To ensure we could reasonably compare the findings from the current study with the prior Skar et al. (2022) investigation when students were in first grade, all the second-grade students in the During COVID-19 cohort in this study were in the same group in the prior investigation.

### COVID-19 Had No Effect on Students’ Writing One Year into the Pandemic

Contrary to our prediction, we did not find statistically significant differences between the Before COVID-19 cohort and the During COVID-19 cohort on any of the three writing measures: writing quality, handwriting fluency, or attitude toward writing. Consequently, by the end of second grade, the Norwegian students who experienced remote instruction as first-graders in 2020 and completed another year of schooling during the pandemic in 2020/2021 wrote as well, had as fluent handwriting, and were just as positive about writing as their peers were at the end of the school year before the pandemic began. In other words, the writing losses observed when the During COVID-19 cohort were in first grade immediately following the end of remote instruction in April, 2020 (see Skar et al., 2022), were not evident 1 year later at the end of second grade even though the pandemic was still underway.

Normally, we would recommend that such findings need to be replicated with other Norwegian children, but the uniqueness of this situation makes such a recommendation implausible. We know of no other researchers in Norway who have undertaken such an analysis in writing, and Norway does not administer writing or other tests to primary grade children as a matter of public policy. This does not mean that additional studies examining the long-term effects of the COVID-19 pandemic on the writing and academic skills of students in other countries are not needed. Given the different ways that individual countries have responded to the SARS-CoV-2 virus educationally and generally, we suspect that not all outcomes will be as positive as the one’s from this investigation.

Why did the learning loss in writing observed immediately after remote instruction in Skar et al. (2022) dissipate a year later in the current study even though the pandemic continued? We cannot directly answer that question, but we do offer some possible explanations. First, Norway implemented remote instruction for a relatively short period of time at the end of the 2019/2020 school year. While this did negatively affect students’ performance on the three writing measures immediately after remote instruction ended in first grade (Skar et al., 2022), outcomes may not have been as positive in the current investigation if remote instruction continued as a national policy into second grade for participating students. With a relatively short period of emergency remote instruction, the return to in-person instruction in schools may have been enough to overcome initial losses in writing that occurred as a result of the move to digital instruction. This may not have happened if Norway had implemented a national policy moving all schools to remote instruction for longer or multiple periods of time. This proposition can be tested for reading and mathematics in OECD countries. Both of these skills are assessed in member countries and there was variability across countries in how much and how often emergency remote instruction occurred. Unfortunately, such a test for writing cannot be implemented because writing is not part of the OECD assessments.

It is also possible that the impact of COVID-19 on Norwegian students’ writing scores dissipated because Norway is an affluent country with a strong educational system where teachers have considerable autonomy (Blikstad-Balas et al., 2022). This may have made it easier for schools and teachers to adjust their instruction successfully to meet the ongoing realities of the pandemic once students had returned to in class instruction. More specifically, teachers and students may have gotten better adjusted to dealing with the disruptive effects of the pandemic over the course of the second-grade school year. Studies are needed that examine how teachers and students cope and successfully address pandemic-related issues. New pandemics will occur in the future (Howard & Howard, 2012), and it is imperative that we better understand how they impact students and teachers.

One final observation concerns the finding that school size predicted students’ fluency with handwriting. It is not readily evident why this was the case. To our knowledge, there are no prior studies demonstrating that students in larger schools are faster at producing handwriting. If such a finding is replicated in future studies, it is important to determine why this is the case. For example, larger schools may emphasize writing more than smaller schools, and there is evidence that handwriting fluency is enhanced by writing more (Graham et al., 1998). It is also possible that larger schools have more resources than smaller ones making it possible for them to place a greater emphasis on handwriting or to purchase materials to teach.

## Limitations

As with all studies, the current investigation had multiple limitations. It was not possible or ethical to assign students to a COVID and non-COVID group. We employed a more natural approach to studying the effects of the pandemic on students’ writing. We compared the writing performance of second-grade students who had completed our assessment the year prior to COVID-19 to the writing performance of second-grade students in the same schools once the pandemic had been ongoing for a little over a year. While we controlled for variance due to school and student variables for the two groups, there was no way to guarantee the Before and During COVID-19 cohorts were identical.

The Before COVID-19 group included 1668 s graders tested in May/June 2019, whereas the During COVID-19 group included 641 s grade students tested in May/June 2021. The Before COVID-19 group included all second-grade consented students who had completed all three writing assessments in 185 schools. The During COVID-19 group included all consented second grade students who completed all three writing assessments from the 185 schools that had been randomly assigned to a no-treatment control condition in a writing intervention study. While there were no statistically detectable differences in the writing performance of the students who received a writing intervention and those who did not (the During COVID-19 group in this study), this does not ensure the findings from this investigation would have remained the same if no writing intervention had been administered and the sample for the During COVID-19 group included untreated students from all 185 schools. This must be considered when interpreting the findings from this study.

We also concentrated our analysis in this study on second-graders. While it is important to study children in other grades, the nature of the current investigation dictated the grade we examined. Our During COVID-19 cohort were derived from an earlier study involving first-grade children (Skar et al., 2022), and we assessed these children’s writing 1 year later when they were at the end of second grade. Not enough time had passed to look at third grade or beyond.

Similarly, our assessment of writing was limited to three writing constructs: quality, handwriting fluency, and attitude toward writing. While each of these assessments measured an important writing construct, they do not assess all students need to know or do as second-grade writers. This must be considered when interpreting the results of our study.

Finally, this study would have been enriched if we had obtained information on how writing was taught by schools to the participating students during emergency remote instruction in and the year following during the ongoing pandemic. This may have helped explain why writing losses observed immediately after remote instruction in April 2020 dissipated a year later at the end of second grade. We could have then examined what aspects of writing instruction best predicted students’ writing performance at the end of second grade. Moreover, we did not collect any information from parents about possible instruction in writing they may have provided to their children or arranged for them to obtain. A study by Blikstad-Balas et al. (2022) with parents raised concerns about the quality of instruction young children received during emergency remote instruction, but we are unaware of any studies that examined Norwegian parents’ role in teaching writing to their children following the relatively short suspension of in-person schooling in Norway. It is possible, therefore, that parents played an instructional role in mitigating the impact of the pandemic on the writing of young Norwegian students.

## Concluding Comment

The findings from this study revealed that the impact of the COVID-19 pandemic on students’ learning, at least for writing, may not be as dire as many predicted (Daniel, 2020; Schleicher, 2020). This is not certain, however, requiring that research continues to be conducted to monitor the educational effects of this pandemic and ones that occur in the future.

## Data Availability

Data set is available upon request to the first author.

## Appendix A

**Flow Chart of Participants**

*Legend*

Data for this investigation originates from a large-scale writing intervention project (Skar et al. (2020)), that took place during the COVID-19-pandemic. Prior to the pandemic, in 2019, students from schools that agreed to participate in the intervention project participated in a baseline measure prior the randomized allocation to arms. In grade 2, 2,276 students were approached and 2,076 (91.2%) consented to participation. Of those students, 80.3% (*N* = 1,668) participated fully (i.e., completed all writing assessments). These students formed the Before COVID-19-group. After the baseline measure was conducted and after schools had been randomized into treatment and control conditions, student from the control condition were tested (*N* = 817). These students were affected by the COVID-19-pandemic and thus formed The During COVID-19-group. Of those students, 78.5% (*N* = 641) participated in the study. Twenty five were eliminated because they had not participated in the first COVID-19-study (Skar et al., 2022), and another 151 were eliminated because they had not completed all writing assessments.

## Footnotes

1 See < https://www.helsedirektoratet.no/veiledere/smittevern-for-skoletrinn-1-7-covid-19/smitteforebyggende-tiltak/nivainndeling-av-smitteverntiltak-trafikklysmodell > for a comprehensive description of this model.

2 We tested this using a multilevel modeling approach. After accounting for the same predictors as in the main analyses of the current paper, the *p*-value for condition was .78 for text quality, .31 for handwriting fluency, and .89 for attitude toward writing.

3 Using a multilevel approach to longitudinal data, we tested if “rating occasion” significantly predicted the score on these 50 texts. This was not the case. The ICC (i.e., the within-text correlation) was .99.

4 https://www.oslomet.no/forskning/forskningsprosjekter/klasserommets-praksisformer-20-ar-etter

5 https://www.utdanningsnytt.no/aktuelt-navn-reform-skole/skal-leie-evalueringa-av-seksarsreformen-vi-ma-sja-pa-kva-som-skjer-i-klasseromma/254413

## References

[CR1] Azoulay, A. (2020). *COVID: 12 education responses.* https://rb.gy/kuckhk

[CR2] Azner A, Sowden P, Bayless S, Ross K, Warhurst A, Pachi D (2021). Home-schooling during COVID-19 lockdown: Effects of coping style, home space, and everyday creativity on stress and home-schooling outcomes. Couple and Family Psychology: Research & Practice.

[CR3] Bates, D., Mächler, M., Bolker, B., & Walker, S. (2015). Fitting linear mixed-effects models using lme4. *Journal of Statistical Software, 67*(1), 1–48. 10.18637/jss.v067.i01

[CR4] Blikstad-Balas, M., Roe, A., Dalland, C. P., & Klette, K. (2022). Homeschooling in Norway during the pandemic-digital learning with unequal access to qualified help at home and unequal learning opportunities provided by the school. In F. M. Reimers (Ed.), *Primary and Secondary Education During Covid-19. Disruptions to Educational Opportunity During a Pandemic* (pp. 177–201). Springer. 10.1007/978-3-030-81500-4_7

[CR5] Camping A, Graham S, Ng C, Aitken A, Wilson J, Wdowin J (2020). Writing Motivation of middle school emergent bilingual students. Reading and Writing: An Interdisciplinary Journal.

[CR6] Chan M, Sharkey J, Lawrie S, Arch D, Nylund-Gibson K (2021). Elementary school teacher well-being and supportive measures amid COVID-19: An exploratory study. School Psychology.

[CR7] Cordeiro C, Castro SL, Limpo T (2018). Examining potential sources of gender differences in writing: *The role of handwriting fluency and self-efficacy beliefs*. Written Communication.

[CR8] Daniel, J. (2020). Education and the COVID-19 pandemic. *Prospectus, 49,* 91–96. https://eenee.eu/wp-content/uploads/2021/06/EENEE_AHQ03_Financing-education-in-the-context-of-COVID-19-2.pdf 10.1007/s11125-020-09464-3 PMC7167396 32313309

[CR9] De Witte, K., & Smet, M. (2021). *Financing education in the context of COVID-19* (EENEE Ad hoc report 03/2021). https://eenee.eu/wp-content/uploads/2021/06/EENEE_AHQ03_Financing-education-in-the-context-of-COVID-19-2.pdf

[CR10] Di Pietro, D., Biagi, G., Costa, P., Karpinski, Z., & Mazza, J. (2020). *The likely impact of COVID-19 on education: Reflections based on existing literature and recent international datasets.* EUR 30275 EN, Publications Office of the European Union, Luxembourg.

[CR11] Eckes, T. (2011). *Introduction to many-facet Rasch measurement: Analyzing and evaluating rater-mediated assessments* (2 ed.). Peter Lang.

[CR12] Ekholm E, Zumbrunn S, DeBusk-Lane M (2018). Clarifying an elusive construct: A systemic review of writing attitude. Educational Psychology Review.

[CR13] Esposito, S., Cotugno, N. & Principi, N. (2021) Comprehensive and safe school strategy during COVID-19 pandemic. *Italian Journal of Pediatrics, 47*(6), 1–4. 10.1186/s13052-021-00960-6 10.1186/s13052-021-00960-6 PMC7794615 33422089

[CR14] Fauzi I, Khusuma I (2020). Teachers’ elementary school in online learning of COVID-19 pandemic conditions. Jurnal Iqra.

[CR15] Graham, S. (in press). Writer(s)-Within-Community model of writing as a lens for studying the teaching of writing. In R. Horrowitz (Ed.), *The Routledge Handbook of International Research on Writing*, Vol. II. Routledge.

[CR16] Graham, S. (2018a). A writer(s) within community model of writing. In C. Bazerman, V. Berninger, D. Brandt, S. Graham, J. Langer, S. Murphy, P. Matsuda, D. Rowe, & M. Schleppegrell, (Eds.), *The lifespan development of writing* (pp. 271–325). National Council of English.

[CR17] Graham, S. (2018b). The writer(s)-within-community model of writing. *Educational Psychologist,**53*, 258–279.

[CR18] Graham S, Berninger VW, Abbott RD, Abbott SP, Whitaker D (1997). Role of mechanics in composing of elementary school students: A new methodological approach. Journal of Educational Psychology.

[CR19] Graham S, Berninger V, Weintraub N, Schafer W (1998). The development of handwriting fluency and legibility in grades l through 9. Journal of Educational Research.

[CR20] Graham S, Kiuhara S, MacKay M (2020). The effects of writing on learning in science, social studies, and mathematics: A meta-analysis. Review of Educational Research.

[CR21] Graham S, Liu K, Bartlett B, Ng C, Harris KR, Aitken A, Barkel A, Kavanaugh C, Talukdar J (2018). Reading for writing: A meta-analysis of the impact of reading and reading instruction on writing. Review of Educational Research.

[CR22] Haelermans C, Jacobs M, van Vugt L, Aarts B, Abbink H, Smeets C, van der Velden R, Wetten S (2021). A full year COVID-19 crisis with interrupted learning and two school closures: The effects on learning growth and inequality in primary education (Research Memorandum 021).

[CR23] Hammerstein S, Kong C, Dreisorner T, Frey A (2021). Effects of COVID-19 related school closures on student achievement – A systematic review. Frontiers in Psychology.

[CR24] Harmey, S., & Moss, G. (2021). Learning disruption or learning loss: Using evidence from unplanned closures to inform returning to school after COVID-19. Educational Review 1–20. 10.1080/00131911.2021.1966389

[CR25] Howard P, Howard J (2012). Pandemic and pedagogy: Elementary school teachers’ experiences of H1N1 influenza in the classroom. Phenomenology & Practice.

[CR26] Hodges, C., Moore, S., Lockee, B., Trust, T., & Bond, A. (2020). The difference between emergency remote teaching and online learning.* Educause Review, 27*, 1–12. https://er.educause.edu/articles/2020/3/the-difference-between-emergency-remote-teaching-and-online-learning

[CR27] Huber, S., Gunther, P., Scneider, N., Helm, C., Schwander, M., Schneider, J., & Pruitt, J. (2020). *COVID-19 und aktuelle Herausforderungen in Schule und Bildung*. https://www.waxmann.com/index.php?eID=download&buchnr=4216

[CR28] König C, Frey A (2022). The impact of COVID-19-related school closures on student achievement—A meta-analysis. Educational Measurement: Issues and Practice.

[CR29] Linacre, J. M. (2018a). *A user's guide to FACETS. Rasch-model computer programs. Program manual 3.80.4*. Winsteps.com.

[CR30] Linacre, J. M. (2018b). *Facets® (version 3.80.4)* [Computer Software]. Winsteps.com.

[CR31] Lorah J (2018). Effect size measures for multilevel models: Definition, interpretation, and TIMSS example. Large-Scale Assess Educ.

[CR32] Monroe, M., & Sherman, E. E. (1996). *Group diagnostic reading aptitude and achievement test*. C. H. Nevins.

[CR33] Norwegian Directorate for Education and Training. (2021). *Konsekvenser av smitteverntiltak i grunnskolen – våren 2021*. https://www.udir.no/tall-og-forskning/statistikk/statistikk-grunnskole/analyser/konsekvenser-av-smitteverntiltak-i-grunnskolen--varen-2021/

[CR34] OECD. (2021). *The state of global education: 18 months into the pandemic.* OECD Publishing. https://www.oecd.org/education/state-of-school-education-one=year-into-covid.htm

[CR35] R Core Team (2020). R: A language and environment for statistical computing. R Foundation for Statistical Computing, Vienna, Austria. http://www.R-project.org/.

[CR36] Reilly D, Neumann D, Andrews G (2019). Gender differences in reading and writing achievement: Evidence from the National Assessment of Educational Progress (NAEP). American Psychologist.

[CR37] Reimers F, Reimers F (2022). Learning from a pandemic: The impact of COVID-19 on education around the world. Primary and secondary education during COVID-19.

[CR38] Schleicher, A. (2020). *The impact of COVID-19 on education: Insights from education at a glance.* OECD Publishing. https://www.oecd.org/education/education-at-a-glance/

[CR39] Slavin R, Madden N, Karweit N, Slavin R, Karweit N, Madden N (1989). Effective programs for students at risk: Conclusions for practice and policy. Effective programs for students at risk.

[CR40] Snijders T, Bosker R (2012). Multilevel analysis: An introduction to basic and advanced multilevel modeling (2nd edition).

[CR41] Skar GB, Graham S, Huebner A (2022). Learning loss during the COVID-19 pandemic and emergency remote instruction first grade students’ writing: A natural experiment. Journal of Educational Psychology.

[CR42] Skar, G. B., Aasen, A. J., & Jølle, L. (2020). Functional writing in the primary years: protocol for a mixed-methods writing intervention study. *Nordic Journal of Literacy Research, 6*(1), 201–216. 10.23865/njlr.v6.2040

[CR43] Skar GB, Aasen AJ, Kvistad AH, Johansen MB (2022). Audience awareness in elementary school students’ texts: Variations within and between Grades 1–3. Writing & Pedagogy.

[CR44] Skar GB, Jølle L, Aasen AJ (2020). Establishing scales to assess writing proficiency development in young learners. Acta Didactica Norge.

[CR45] Skar GB, Kvistad AH, Johansen MB, Rijlaarsdam G, Aasen AJ (2022). Identifying texts in the warning zone: Empirical foundation of a screening instrument to adapt early writing instruction. Writing & Pedagogy.

[CR46] Skar GB, Lei P-W, Graham S, Aasen AJ, Johansen MB, Kvistad AH (2022). Handwriting fluency and the quality of primary grade students’ writing. Reading and Writing.

[CR47] UNESCO. (2020). *COVID-19 impact on education.* https://en.unesco.org/COVID-1919/educationresponse.

[CR48] Walberg H, Ethington C (1991). Correlates of writing performance and interest: A U.S., National assessment study. Journal of Educational Research.

[CR49] Winthrop, R. (2020). COVID and school closures: What can countries learn from past closures? *Brookings*. https://rb.gy/ctwu4n

